# Carcinosarcoma of the Uterus: A Study From the Surveillance Epidemiology and End Result (SEER) Database

**DOI:** 10.7759/cureus.10283

**Published:** 2020-09-06

**Authors:** Noor Nama, Frederick D Cason, Subhasis Misra, Shaikh Hai, Veronica Tucci, Furqan Haq, Joseph Love, Asad Ullah, Pete Peterson, Fernando F Grishko, Wazir Akbar, Khalida Khaliq, Abdul Waheed

**Affiliations:** 1 Obstetrics and Gynaecology, Bolan Medical College, Quetta, PAK; 2 Surgical Oncology, San Joaquin General Hospital, Stockton, USA; 3 Surgery, Brandon Regional Hospital, Brandon, USA; 4 Surgery, Hospital Corporation of America East Florida Graduate Medical Education Consortium, Plantation, USA; 5 Emergency Medicine, Hospital Corporation of America East Florida Graduate Medical Education Consortium, Plantation, USA; 6 Medicine, Hospital Corporation of America West Florida, Tampa, USA; 7 Surgery, Bayonet Point Regional Medical Center, Hudson, USA; 8 Pathology, Medical College of Georgia - Augusta University, Augusta, USA; 9 Surgery, University of South Florida Health, Tampa, USA; 10 Surgery, Hospital Cardiológico Infantil Latinoamericano, Havana, CUB; 11 Neurology, Bolan Medical College, Quetta, PAK; 12 Psychiatry and Medicine, North Tampa Behavioral Health, Tampa, USA; 13 Surgery, Sandeman Provincial Hospital, Quetta, PAK

**Keywords:** endometrioid adenocarcinomas, uterine cancer, carcinosarcoma, seer

## Abstract

Background: Uterine cancer (UC) is one of the leading gynecologic neoplastic disorders in the United States (US), of which over 80% are endometrioid adenocarcinomas (EA). In contrast to EA, carcinosarcoma (CS) of the uterus is a sporadic and highly malignant tumor, phylogenetically containing both epithelial and mesenchymal histologic elements. This study sought to analyze demographic, pathological retrospectively, and survival characteristics of a large cohort of CS patients compared to EA patients to identify prognostic factors and treatment approaches that may improve the current clinical management of CS patients.

Methods: Demographic and clinical data were abstracted from 88,530 patients diagnosed with uterine malignancy from the Surveillance, Epidemiology, and End Results (SEER) database for 38 years (1973-2010). Extracted variables were analyzed using the Chi-square test, paired t-test, and multivariate analysis, while Kaplan-Meier functions were used to compare survival between groups. Statistical analyses were performed with IBM Statistical Product and Service Solutions (SPSS^©^), version 20.2 (IBM Corp., Armonk, NY).

Results: A total of 3,706 cases of CS comprised 38.2% of uterine sarcomas (n=9,702), and 4.1% of uterine cancers overall (n=88,530). EA made up 88.6% (n=78,481) of all uterine cancers. CS patients presented later in life (68.3±11.5 years) than EA (61.9±12.5 years). 65.2% of CS and 77.8% of EA occurred in Caucasians. The incidence (per million) of EA was higher in Caucasians compared to African-Americans (AA) (41% vs. 26.8%), while the incidence of CS was higher among AA than Caucasians (4% vs. 1.9%, p<0.001). 33.4% of CS was poorly differentiated at presentation, compared to 13.1% of EA. 27.8% of CS patients presented with a distant disease compared to only 4.7% of EA patients. 29.9% of AA patients with CS presented with metastatic disease, compared to 28.2% of Caucasian patients (p<0.001). Mean survival for CS patients (6.6±0.2 years) was significantly lower than that of EA patients (17.7±0.7 years, p<0.001), and AA CS patients had significantly lower survival than Caucasians CS patients (4.5±0.4 years vs. 7.1±0.3 years, p<0.001). CS patients treated with combined surgery and radiotherapy had the highest survival (9.4±0.5 years, p<0.001), while EA patients treated with surgery alone had the highest survival (20.4±1.2 years, p<0.001). Survival among AA CS patients treated with combination therapy was significantly inferior compared to Caucasians (6.5±0.6 years vs. 9.8±0.5 years, p<0.001). Multivariate analysis identified CS histology (odds ratio [OR] 1.9, CI=1.7-2.1), AA race (OR 1.3, CI=1.2-1.4), age over 40 (OR 3.4, CI=2.9-4.1), undifferentiated grade (OR 3.0, CI=2.6-3.4), and distant metastases (OR 6.2, CI=5.8-6.8) as independently associated with increased mortality (p<0.005). The use of radiotherapy in CS patients was independently associated with decreased mortality (OR 0.1, CI=0.02-0.6, p<0.005).

Conclusions: Uterine CS is a highly malignant tumor with a significantly worse prognosis than EA. AA has a considerably higher CS incidence compared to EA. Moreover, AA CS had higher tumor grades, higher rates of metastatic disease, and experienced significantly lower overall survival compared to Caucasians despite receiving similar therapy. Primary radiotherapy or combination radiotherapy confers a survival advantage to AA uterine CS patients.

## Introduction

Endometrioid adenocarcinomas (EA) comprise up to 88% of all uterine cancers and are the most common gynecologic malignancy in developed countries [1]. It is estimated that 12,600 women will die from uterine cancer in 2020, making it the sixth most common cause of cancer mortality among women in the United States [1]. Uterine sarcomas comprise endometrial stromal sarcomas, leiomyosarcomas, carcinosarcomas, and undifferentiated sarcomas. Uterine carcinosarcoma (CS), or mixed Mullerian tumor (mMT), is a rare and highly aggressive neoplasm comprising less than 5% of uterine corpus malignancies but accounting for 16.4% of all deaths from uterine cancer [1-3]. With an annual incidence of 1.7 per 100,000 women, CS is a biphasic tumor that is histologically characterized by cellular elements derived from both ectodermal and mesodermal origins, i.e., carcinomatous (epithelial) and sarcomatous (mesenchymal) components. Three theories exist as to its source: (i) the “collision theory” hypothesizes that CS is the product of individual epithelial and mesenchymal tumors in close proximity that “collide” or grow into one another, forming a distinct, new cancer entity; (ii) the “combination theory” proposes that CS originates from a conventional epithelial precursor stem cell which undergoes bi-directional differentiation into epithelial and mesenchymal tissue; and (iii) the most widely accepted theory of “conversion” suggests that CS is formed when either a pre-existing carcinoma undergoes sarcomatous transformation or a pre-existing sarcoma undergoes epithelial differentiation [4].

Risk factors for CS are similar to those for endometrioid adenocarcinomas (EA). They include advanced age, obesity, nulliparity, and exposure to exogenous estrogen as well as selective estrogen receptor modulators (SERM), like adding tamoxifen [4-6]. The main symptoms of CS include abnormal menses, post-menopausal bleeding, abdominal mass or discomfort, urinary symptoms, and infertility [5-7]. The International Federation of Gynecology and Obstetrics (FIGO) recommend that CS be staged equivalently to EA, and clinic-pathological disease stage at the time of diagnosis is the most important factor affecting prognosis, notably increased tumor burden and the amount of myometrial and vascular invasion [6-8]. However, unlike EA, up to 30% of patients can have extra-uterine disease at the time of presentation, resulting in a relatively weak prognosis [1-6]. The 5-year overall survival rate of CS is 30%-45% for patients with stage I or II diseases, and 0%-10% for stage III or IV disease [6-8].

The mainstay of treatment for both EA and CS is surgery, involving total abdominal hysterectomy (TAH), bilateral salpingo-oophorectomy (BSO), and lymph node dissection (LND), with or without combination chemoradiation [9-14]. Despite this aggressive surgical approach, locoregional recurrence rates occur in up to 60% [14]. Substantial data documenting survival rates for CS patients is limited and consists primarily of a few population-based studies involving small groups of patients from high volume cancer centers [15-21]. As a result of the insufficient data in CS outcome, demographic and clinical factors remain poorly understood. However, recent case reports are emerging of CS being a part of or mistaken for myomas, making clinical knowledge of the characteristics and risk factors for CS essential, and especially crucial for gynecologists to avoid improper management or inadvertent cancer dissemination during surgery [22-25]. The current study examines a large modern cohort of primary CS patients from the Surveillance, Epidemiology, and End Result (SEER) database over 38 years to better understand the temporal associations demographic and management trends and to determine unique factors predictive of clinical outcomes. 

## Materials and methods

Data were extracted from the SEER database between 1973 and 2010. SEER Stat software using registries from Alaska Native Tumor Registry, Arizona Indians, Cherokee Nation, Connecticut, Detroit, Georgia Center for Cancer Statistics, Greater Bay Area Cancer Registry, Greater California, Hawaii, Iowa, Kentucky, Los Angeles, Louisiana, New Jersey, New Mexico, Seattle-Puget Sound, and Utah were used. A total of 88,530 patients with all subtypes of uterine cancer were identified and exported to IBM Statistical Product and Service Solutions (SPSS©), version 20.2 (IBM Corp., Armonk, NY). Patients with a primary diagnosis of EA or CS were identified to form the study cohort, using the SEER International Classification of Disease for Oncology (ICD-O-3) codes based on histological subtypes: “Endometrioid Adenocarcinoma” (8380) and “Carcinosarcoma” (8981, 8982) of the uterus. Demographic and clinical data extracted included age, gender, race, tumor histology, stage, grade, and type of treatment received (surgery, radiation, both or unknown/no treatment). The final endpoints were 1-, 2-, and 5-year overall survival, and also treatment-specific survival. The Chi-square test analyzed the categorical variables in the dataset. At the same time, all the continuous variables were analyzed with the Student t-test and also the analysis of variance (ANOVA). To calculate the odds ratio (OR) to find the independent factors affecting survival, the multivariate analysis was performed using the “backward elimination wald” method. Missing and unknown data were excluded from the multivariate analysis. To compare the long term survival, the Kaplan-Meier analysis was used. Statistical significance was accepted at p<0.05.

## Results

Demographic data

A total of 82,187 cases of EA and CS were identified from the SEER database over 38 years (1973-2010), of which 78,481 (95.5%) had EA and 3,706 (4.5%) had CS (p<0.005) (Table 1). The mean overall age at diagnosis for all uterine cancer patients was 62.2±12.6 years; however, CS patients presented later in life (68.3±11.5 years) compared to EA patients (61.9±12.5 years) (p<0.001).

**Table 1:** Demographic Profiles of 78,481 patients with Endometrioid Adenocarcinoma and 3,706 patients with Carcinosarcoma of the Uterus from the Surveillance Epidemiology and End Result (SEER) Database (1973-2010). Abbreviations: N = number; SD = standard deviation; Diff = Differentiated.*p value: statistical significance, < 0.05; ^age-adjusted incidence for the years 1973-2010

Variables	Overall	Carcinosarcoma	Endometrioid Adenocarcinoma	p-values
N (%)	82,187	3,706 (4.5)	78,481 (95.5)	0.025
Age, (Mean ± SD)	62.19±12.6	68.32±11.5*	61.9±12.5	<0.001
RaceN(%)				
Caucasian	63,438 (77.2)	2,417 (65.2)	61,021 (77.8)*	<0.001
Hispanic	6,842 (8.3)	312 (8.4)	6,530 (8.3)	<0.001
African American	5,487 (6.7)	730 (19.7)*	4,757 (6.1)	<0.001
Other	5,912 (7.2)	237 (6.4)	5,675 (7.2)	0.03
Unknown	508 (0.6)	10 (0.3)	498 (0.6)	0.05
Incidence (per million)^				
All races	42.0	1.9	40.1	<0.001
Caucasian	42.8	1.8	41.0*	<0.001
African American	30.8	4.0*	26.8	<0.001
Other	40.3	1.5	38.8	<0.001

Ethnographically, the majority of both types of uterine cancer occurred in Caucasians (63,438 patients; 77.2%). There were 61,021 Caucasian EA patients (77.8%) and 2,417 Caucasian CS patients (65.2%) (p<0.001). With regards to EA, Hispanics (6,530 patients, 8.3%) were the next most common group, followed by African Americans (4,757 patients, 6.1%). In contrast, AA was the second largest ethnic group affected by CS (730 patients; 19.7%), followed by Hispanics (312 patients; 8.4%) (p<0.001).

The overall incidence (per million) of both cancers was highest among Caucasians (42.8). The incidence of EA was significantly higher among Caucasians (41 in Caucasians vs. 26.8 in AA) (p<0.001), while the incidence of CS was significantly higher among AA (1.9 in Caucasians vs. 4.0 in AA) (p<0.001).

Tumor characteristics

Histologically, a majority of EA were well-differentiated (35,308 patients; 45%) or moderately-differentiated (25,094 patients; 32%) compared to CS. The majority of CS were poorly differentiated (1,239 patients; 33.4%) or undifferentiated (657 patients; 17.7%). Histologic grade was unknown in 8,350 (10.2%) patients (p=0.05).

When comparing disease stage at presentation, the majority of EA patients had localized disease (60,167 patients; 76.7%), while the majority of CS patients presented with the distant disease (1,031 patients; 27.8%) (p<0.001). Among AA patients specifically, 29.9% presented with stage IV disease, compared to only 28.2% of Caucasian CS patients, p<0.001. Disease stage was not determined in 2,157 (2.6%) patients (p=0.06) (Table 2).

**Table 2:** Tumor Characteristics of 78,481 patients with Endometrioid Adenocarcinoma and 3,706 patients with Carcinosarcoma of the Uterus from the Surveillance Epidemiology and End Result (SEER) Database (1973-2010). Abbreviations: N = number; SD = standard deviation; Diff = Differentiated.*p value: statistical significance, < 0.05; ^age-adjusted incidence for the years 1973-2010

Variables	Overall	Carcinosarcoma	Endometrioid Adenocarcinoma	p-values
GradeN(%)				
Well Differentiated	35,354 (43.0)	46 (1.2)	35,308 (45.0)*	<0.001
Moderately Diff.	25,205 (30.7)	111 (3.0)	25,094 (32.0)*	<0.001
Poorly Diff.	11,555 (14.1)	1,239 (33.4)*	10,316 (13.1)	<0.001
Undifferentiated	1,723 (2.1)	657 (17.7)*	1,066 (1.4)	<0.001
Unknown	8,350 (10.2)	1,653 (44.6)	6,697 (8.5)	0.05
Stage N (%)				
Localized	61,612 (75.0)	1,445 (39.0)	60,167 (76.7)*	<0.001
Regional	13,697 (16.7)	1,058 (28.5)*	12,639 (16.1)	<0.001
Distant	4,721 (5.7)	1,031 (27.8)*	3,690 (4.7)	<0.001
Unstaged	2,157 (2.6)	172 (4.6)	1,985 (2.5)	0.06

Treatment

Surgery alone was the most common treatment for both types of uterine cancer (58,205 patients; 70.8%), followed by combination of surgery and radiotherapy (19,506 patients; 23.7%) (Table 3). A total of 56,175 (71.6%) EA patients and 2,030 (54.8%) CS patients underwent primary surgical therapy (p<0.001). More CS patients underwent combination surgery and radiotherapy (1,256 patients; 33.9%) compared to EA (18,250 patients; 23.3%) (p<0.001). A total of 3,185 patients (3.9%) were untreated, with significantly more CS patients being untreated (n=260; 7.0%) compared to EA (n=2,925; 3.7%) (p<0.001). Among patients receiving no treatment, 9.7% were AA, 6.7% were Caucasian, 6.4% were Hispanic and 4.2% were Asian/Pacific Islanders (p<0.001). Treatment was unknown in 313 patients (0.4%) (p=0.03). 

**Table 3:** Treatment and Survival Outcomes of 78,481 patients with Endometrioid Adenocarcinoma and 3,706 patients with Carcinosarcoma of the Uterus from the Surveillance Epidemiology and End Result (SEER) Database (1973-2010). Abbreviations: N = number; SD = standard deviation; Diff = Differentiated.*p value: statistical significance, < 0.05; ^age-adjusted incidence for the years 1973-2010

Variables	Overall	Carcinosarcoma		Endometrioid Adenocarcinoma		p-values
Treatment N(%)						
No treatment	3,185 (3.9)	260 (7.0)*		2,925 (3.7)		<0.001
Surgery only	58,205 (70.8)	2,030 (54.8)		56,175 (71.6)*		<0.001
Radiation only	978 (1.2)	138 (3.7)		840 (1.1)		<0.001
Both surgery and radiation	19,506 (23.7)	1,256 (33.9)*		18,250 (23.3)		<0.001
Unknown	313 (0.4)	22 (0.6)		291 (0.4)		0.03
Mean Survival (years)	16.88±0.3	6.63±0.24		17.69±0.68		0.025
Survival by treatment (years±SD)						
No treatment		0.74±0.11		7.21±0.24		<0.001
Surgery only		5.83±0.31		20.38±1.16*		<0.001
Radiation only		2.80±0.68		3.50±0.24		<0.001
Both surgery and radiation		9.38±0.45*		15.24±0.46		<0.001
Overall Survival (Cumulative %)						
1-year 2-year 5-year			47*		95	<0.001
			38*		88	<0.001
			14*		62	<0.001

Outcomes

**Figure 1:**
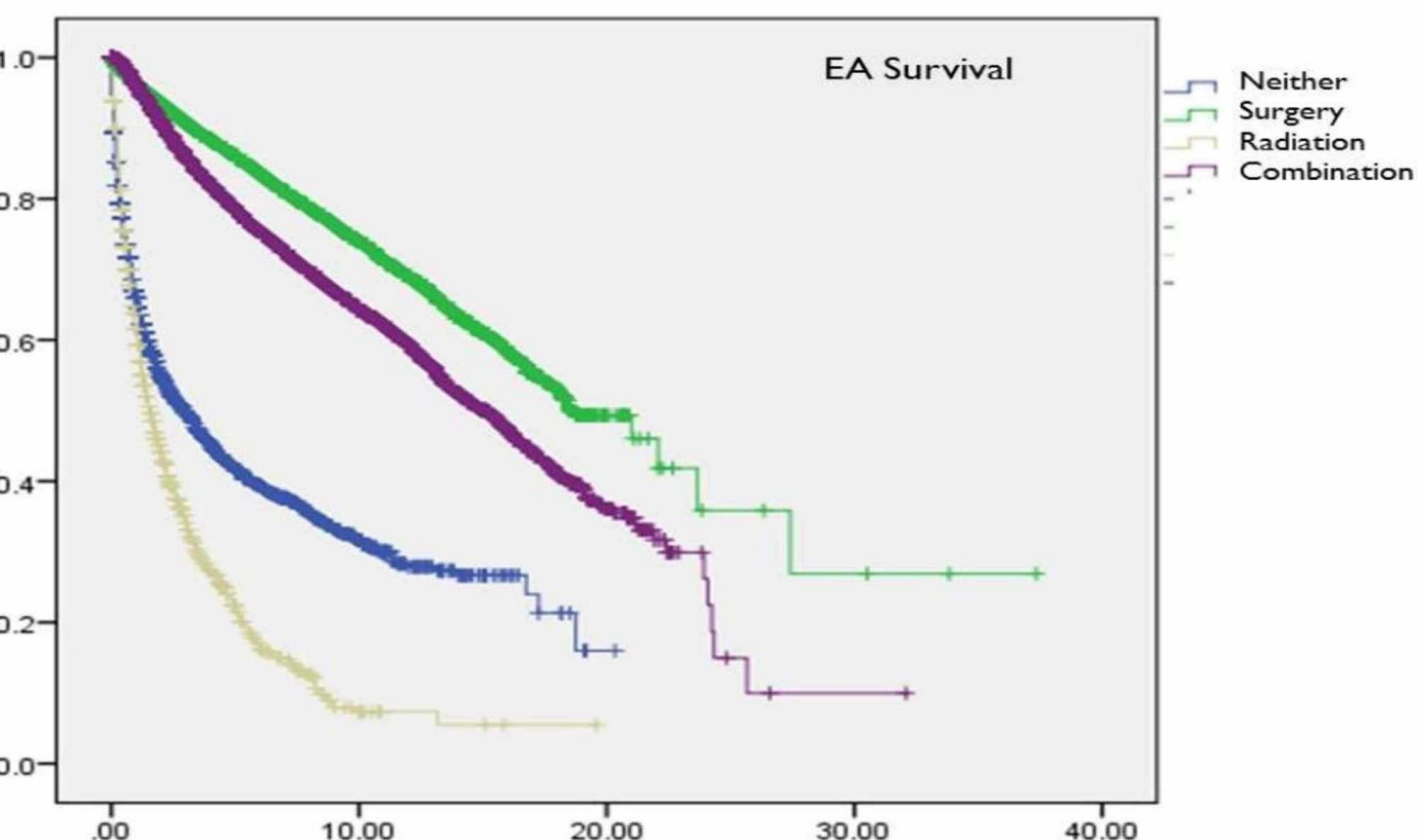
Kaplan Meier Survival Curves for 78,481 patients with Endometrioid Adenocarcinoma from the Surveillance Epidemiology and End Result (SEER) Database (1973-2010), Stratified by Treatment Modality Abbreviations: EA = Endometrioid Adenocarcinoma

**Figure 2:**
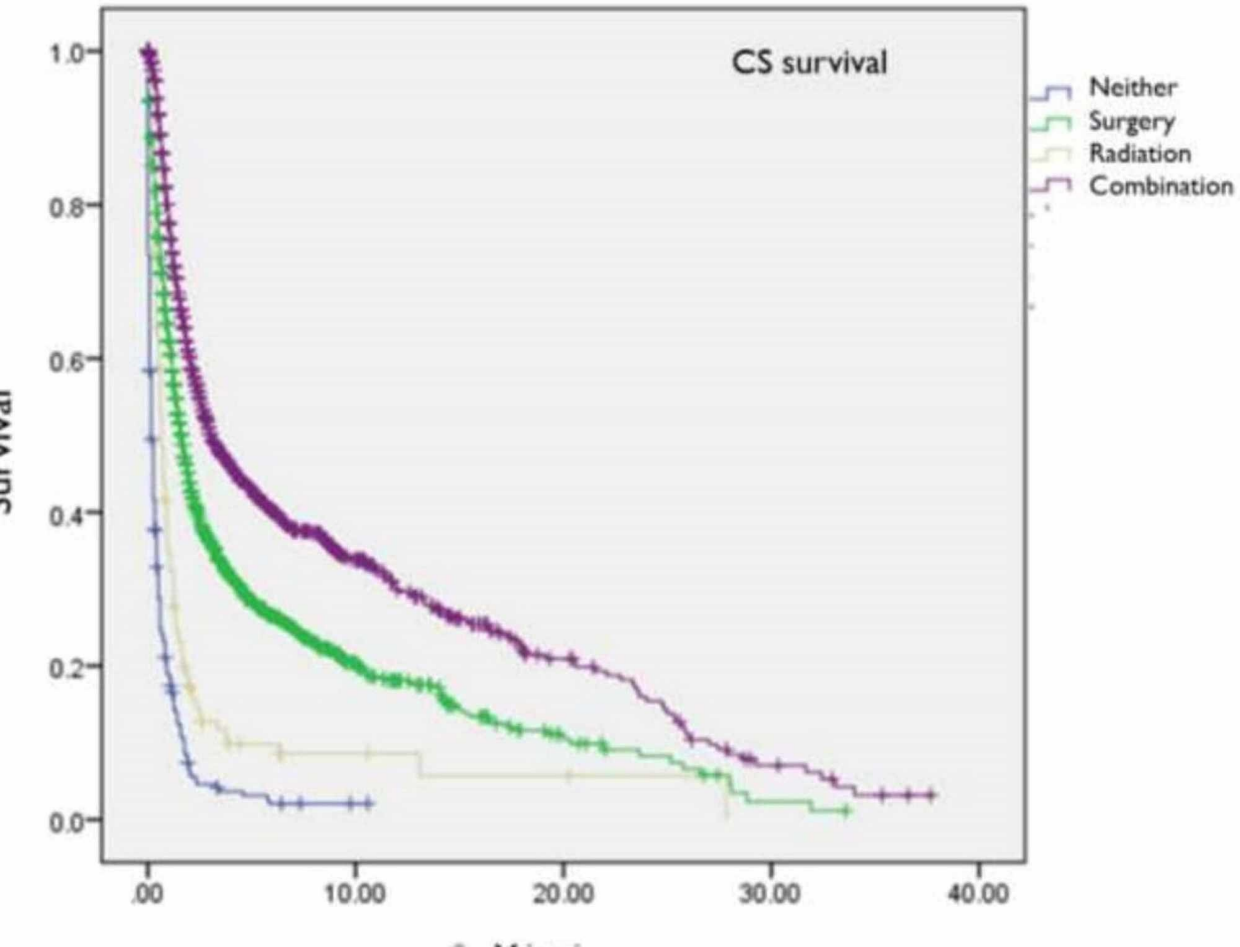
Kaplan Meier Survival Curves for 3,706 patients with Carcinosarcoma of the Uterus from the Surveillance Epidemiology and End Result (SEER) Database (1973-2010), Stratified by Treatment Modality Abbreviations: CS = Carcinosarcoma

The mean overall survival for CS patients was significantly lower (6.6±0.2 years) than that of EA patients (17.7±0.7 years, p<0.005) (Table 3). The 1-, 2-, and 5-year overall survival for CS patients were 47%, 38%, and 14% compared to 95%, 88%, and 62% for EA patients (p<0.001). Survival among EA patients treated with surgery alone (20.4±1.2 years) was slightly higher than EA patients treated with combination therapy (15.2±0.5 years, p<0.001) (Figure 1). In contrast, CS patients treated with surgery and radiotherapy (9.4±0.5 years) had increased survival compared to those treated with surgery only (5.8±0.3 years, p<0.001) (Figure 2). Mean survival was significantly worse among AA patients with CS compared to Caucasians (4.5±0.4 vs. 7.1±0.3, p<0.001). Moreover, overall survival was worse among AA CS patients receiving combination therapy (6.5±0.6 vs. 9.8±0.5, p<0.001) or surgery alone compared to Caucasians (4.3 ± 0.57 vs. 6.3 ± 0.37, p<0.001). For EA, while there was similarly inferior overall and treatment-specific survival for AA compared to Caucasians, these observations were not statistically significant (Table 3, Figure 1 and Figure 2) 

Multivariate analysis

Multivariate analysis identified CS tumor histology (OR 1.9, CI=1.7-2.1), AA race (OR 1.3, CI=1.2-1.4), age over 40 (OR 3.4, CI=2.9-4.1), undifferentiated grade (OR 3.0, CI=2.6-3.4), and presence of distant metastases (OR 6.2, CI=5.8-6.8) as independently associated with increased mortality (p<0.005). The use of radiotherapy in CS patients was independently associated with decreased mortality (OR 0.1, CI=0.02-0.6, p<0.005). 

## Discussion

Uterine cancer is one of the leading gynecologic malignancies in the United States and carries a lifetime risk of 2.6% for all women [1]. EA accounts for 88% of all uterine malignancies, while CS accounts for less than 5% [1-4]. In the current study, EA and CS accounted for 88.9% and 4.1% of uterine malignancies, respectively. The rarity of CS may be attributed to its unique histologic make-up and its poorly understood pathogenesis, which has challenged clinicians since it was first described in a 1940 case report by Dixen and Dockerty [1-3]. Initially thought to be a uterine sarcoma, CS has been reclassified as a biphasic tumor with an admixture of carcinomatous and sarcomatous histologic components, and has been managed similarly to EA over the last three decades [1-4]. Despite similarities in symptomology and staging, however, the current study highlights several key differences between CS and EA.

Unlike EA, the incidence of CS is over two times higher in AA than in Caucasians in the current study. Several previous reviews have concluded that CS is up to twice as prevalent among AA women compared to Caucasians at every age group and disease stage [8,14-16]. A population-based outcomes review of all uterine malignancies over six years from the National Cancer Institute by Sherman et al. identified 897 CS cases. It showed a significantly higher overall incidence rate of CS among AA women compared to Caucasians (1.82 vs. 0.78 per 100,000) [14]. These findings suggest that AA women may benefit from screening programs for CS compared to Caucasian women. Although there are no specific tumor markers or biochemical tests to detect CS, transvaginal ultrasound or pelvic MRI in AA women at higher risk (age over 40, as per the multivariate analysis in this study) might be a feasible option to increase early detection and lower mortality.

Like high-grade EA, the management of CS is primarily surgical, with increased survival demonstrated in patients undergoing radical resection and removal of all grossly visible tumors [22]. The role of primary radiotherapy in CS remains debated, although several studies have shown improved local-control, disease-specific survival, and mortality rates [8-11, 22]. In the current study, CS patients had the highest overall survival when treated with combined surgery and radiotherapy, as opposed to single-modality treatment alone. In a retrospective study assessing disease progression in 50 CS patients over 15 years, Yu et al. reported that patients who received adjuvant radiotherapy 30 days after surgical resection had no loco-regional progress at 11 months after surgery, while 60% of patients receiving no radiation experienced disease recurrence during the same time period [24]. In a 10-year retrospective analysis of 44 CS patients, Park et al. found that patients with combined surgery and radiotherapy had a 10-year recurrence-free survival of 30% and 10-year overall survival of 48%, which is significantly better than historical 10-year survival rates for CS patients treated with surgery alone (range 5%-15%) [4,5-9,22-23]. These findings suggest a recommendation that the treatment of CS should consist of early initiation of adjuvant radiotherapy within 30 days of resection to decrease recurrence rates and indicate that a prospective randomized controlled trial comparing various types of adjuvant radiotherapy in the treatment of CS should be undertaken. Of note, the National Comprehensive Cancer Network® (NCCN®) guideline for CS states that the role of adjuvant radiotherapy is controversial since while it has demonstrated improvement in local, regional pelvic control, no visible improvement in overall survival has been shown [26]. 

The current study identified significantly decreased 1-, 2- and 5-year survival in CS compared to EA. Bansal et al. similarly reported that CS had a significantly worse overall survival compared to EA at all stages of disease in a population-based outcomes study involving 3,000 CS patients from 1988 to 2004 (overall survival of 60% for EA vs. 35% for CS) [4]. The poor outcomes in CS may be attributed to its tendency to present with a more histologically aggressive and advanced disease compared to EA, as the current study found that a majority of CS tended to be poorly differentiated, undifferentiated, and to have metastases at presentation. These findings again emphasize the potential benefit for the institution of a rigorous screening program for high-risk CS patients, with the early initiation of aggressive therapy when cancer is detected [4].

Inferior clinical outcomes were observed among AA CS patients compared to Caucasians, which can be at least partially explained by the observation that AA CS patients presented with more advanced disease and exhibited inferior response rates to therapy compared to Caucasians. Bansal et al. noted similar findings in their study of 8,986 women with uterine carcinomas and sarcomas in which they reported that AA women with CS were 19% more likely to die from their tumors than Caucasian women (hazard ratio 1.19) [4]. Sherman et al. analyzed the clinical outcomes of 897 CS patients from the National Cancer Institute over 10 years and similarly reported significantly worse survival among AA patients compared to Caucasians even after stratification by stage, grade, and age (mortality rate of 16.43% in AA vs. 9.63% in Caucasians) [14]. This suggests the need for increased awareness among primary clinicians of the inferior survival and worse prognosis of CS in AA patients, to encourage active participation in CS screening programs, and quick earlier visits to their primary care provider if and when symptoms arise. Gynecologists should also be better educated on the poor prognosis of CS among AA to provide adequate education to their AA patients.

In the current study, 9.7% of AA with CS received no treatment, more than any other racial group, despite evidence for improved survival where combination surgery and radiotherapy is provided. Hicks et al. also previously noted an apparent racial disparity in the treatment of uterine cancer in their retrospective review of 52,307 Caucasian and 3,226 AA women with EA from the National Cancer Database over six years, noting that AA women were treated with surgery far less frequently and, among surgically treated patients with advanced stages of the disease, AA received adjuvant radiotherapy significantly less often than Caucasian patients. Besides, similar to the current study, they also reported decreased survival among AA patients who eventually did receive equivalent therapy [15]. Several authors have attributed this apparent discrepancy to AA CS patients having limited access to healthcare given the relatively weak socioeconomic status of a large portion of the AA community, which resulted in delayed disease recognition and management [17-20]. Socioeconomic factors contributing to the poor prognosis of AA patients include lower household income, higher unemployment, poor neighborhood, lower education level, and poor knowledge of health [15-20]. The funding allowed facilitating efforts at detecting and managing CS in public health insurance programs like Medicaid, targeting older, high-risk individuals who may overcome some of these barriers to adequate health care for these populations. Moreover, patient and clinician education regarding CS incorporated at community health centers catering to economically disadvantaged and uninsured patients may also be of value. Finally, private insurance companies should be encouraged to provide coverage for the management of CS in high-risk enrollees to ensure that there is no racial inconsistency in health care [18].

The clinical understanding of CS behavior is slowly evolving. Several reviews have suggested a possible genetic predisposition in AA towards more aggressive, treatment-resistant forms of this tumor [14-21]. Additionally, Naaman et al. demonstrated an increased number of co-existing malignancies with CS, most often breast carcinoma and colon adenocarcinoma, suggesting that CS may share a common genetic locus with other cancers and may represent one component of a yet to be elucidated tumor syndrome [6]. Progress towards establishing the role of molecularly alterations in the pathogenesis of CS is slow; however, due to the low prevalence of this malignancy and the limited survival of most affected patients [6].

There are several limitations of this study, which should be understood in the context of retrospective data extraction from large databases. Though highly accurate, the SEER database did not correctly code for essential clinical variables such as socioeconomic status, tumor depth, and size. Though radiation and chemotherapy data is available in the SEER database yet, the dose of the therapies is missing. Despite all these limitations, the SEER database provides enough information to generalize the results to the general population.

## Conclusions

Uterine CS is a highly malignant tumor that has a significantly higher incidence among AA patients compared to EA. Primary or combination radiotherapy confers a survival advantage in uterine CS. AA patients with CS have more top-grade tumor and more commonly present with distant disease, which is associated with a decreased overall survival compared to Caucasians even when receiving comparable therapy. AA patients may benefit more from rigorous CS screening programs utilizing transvaginal ultrasound or pelvic MRI, particularly in those over 40. If detected, CS should be managed with surgical resection followed by early initiation of adjuvant radiotherapy to decrease recurrence rates. Perhaps the most critical effort to reduce morbidity and mortality among AA CS patients is to promote an increased awareness by the direct patient and clinician education about CS, to encourage the high-risk patient population to actively participate in CS screening programs, promote early primary care visits if symptoms arise, and educate on available clinical trials. To treat patients with limited health care access and poor socioeconomic status, funding should be allocated for the detection and management of CS in both private and public health insurance programs to avoid any racial discrepancies in treatment. Additionally, patient and clinician education regarding CS should be improved at community health centers catering to poor and uninsured patients. Finally, prospective randomized controlled trials focusing on the role of adjuvant radiotherapy in CS patient is desperately needed. 
